# The changes in peripheral blood Th17 and Treg ratios in Hashimoto’s thyroiditis are accompanied by differential PD-1/PD-L1 expression

**DOI:** 10.3389/fendo.2022.959477

**Published:** 2022-08-25

**Authors:** Jun Fang, Lei Yu, Lan-Gen Zhuang, Xiao-Yan Pei, Qiong Wang, Guo-Xi Jin

**Affiliations:** Department of Endocrinology, The First Affiliated Hospital of Bengbu Medical College, Bengbu, China

**Keywords:** PD-1, PD-L1, Hashimoto’s thyroiditis, T helper 17 cells, T regulatory cells

## Abstract

**Objective:**

The aim of this study was to analyze the percentages of T helper 17 cells (Th17s) and T regulatory cells (Tregs) in autoimmune Hashimoto’s thyroiditis (HT), and the expression of the checkpoint molecules programmed death receptor 1/programmed death ligand 1 (PD-1/PD-L1) on these cells.

**Methods:**

This is a case-control study involving 53 initially diagnosed HT patients (HT group) and 21 normal controls (NC group). The peripheral blood mononuclear cells from the individuals of the two groups were isolated and restimulated *ex vivo*; the percentage of Th17s, Tregs, PD-1^+^ Th17s, PD-L1^+^ Th17s, PD-1^+^ Tregs, and PD-L1^+^ Tregs was assessed by flow cytometric analysis.

**Results:**

(1) The percentage of Th17s in the peripheral blood of the HT group was significantly higher than that of the NC group [(6.38 ± 1.32)% versus (3.12 ± 0.66)%; t = 14.110, P < 0.001], while the percentage of peripheral blood Tregs was significantly lower [(3.82 ± 1.48)% versus (5.61 ± 1.60)%; t = −4.599, P < 0.001]. (2) HT patients’ Th17s expressed PD-1 at a significantly lower frequency than their counterparts in the NC [(6.46 ± 2.77)% versus (18.51 ± 3.96)%; t = −14.842, P < 0.001], while no difference was observed for PD-L1 between the two groups. (3) In contrast, both PD-1 and PD-L1 were expressed at significantly higher frequency on HT patients’ Tregs than on NC [respectively: (17.01 ± 3.04)% versus (10.23 ± 2.77)%; t = 8.850, P < 0.001 for PD-1; (16.60 ± 9.58)% versus (11.36 ± 10.14)%; t = 2.089, P < 0.005, for PD-L1].

**Conclusion:**

(1) The increased percentage of Th17s and decreased percentage of PD-1^+^ Th17s in the HT group suggest that a loss of control on Th17 activity through the checkpoint inhibitory axis PD-1/PD-L1 may participate in disease pathogenesis. (2) While the decreased percentage of Tregs in HT patients may explain a lack of regulatory functions able to prevent the autoimmune destruction of the thyroid, the significance of the increased frequency of Tregs expressing PD-1 and PD-L1, previously reported to boost Tregs differentiation, remains to be established. Elucidating this apparent contradiction may reveal important mechanisms underlying HT pathogenesis.

## Introduction

Hashimoto’s thyroiditis (HT) is a common organ-specific autoimmune thyroid disease (AITD) characterized by T cell and antibody-mediated cytotoxicity (1) and with a global incidence increasing year by year. HT specific pathogenesis remains unknown, but a large body of evidence suggests underlying immune dysfunctions (2, 3). The traditional theory holds that interferon-γ (IFN-γ) and tumor necrosis factor-α (TNF-α), which are produced by T helper 1 cells (Th1s) recruited within the thyroid tissue, induce thyrocytes to release CXCL10, which is responsible for initiating and perpetuating of the autoimmune process (4, 5). Studies showed that the CD4^+^ T helper 17 cells (Th17s), producing IL-17 and other pro-inflammatory cytokines, play a major role in the pathogenesis of HT (6–8). IL-17 can act on a broad range of cells to induce the expression of cytokines (e.g., IL-6,IL-8,GM-CSF,and G-CSF), chemokines as CXCL10 by activating the MAPK pathway, thus recruiting neutrophils to produce inflammatory responses to extracellular antigens (9–11). In addition, a decreased number or impaired function of T regulatory cells (Tregs) is strongly correlated with thyroiditis (12, 13). Previous research (14) has confirmed that the loss of Treg function induced by CD25 antibody in IL-17 knockout mice leads to thyroiditis dominated by Th1s. Th17/Treg imbalance is particularly relevant to the occurrence and progression of autoimmune diseases (15, 16). Yet, the exact determinants of CD4^+^ T cell differentiation into Th17 or Treg cells and the mechanisms leading to imbalanced ratio or functions of these cells are still unknown.

The interactions between the immune checkpoint protein programmed cell death-1 (PD-1, also known as CD279) and its ligands programmed cell death-ligand 1 (PD-L1, also known as CD274) and programmed cell death-ligand 2 (PD-L2, also known as CD273) (17–19) are important negative regulators of T cell proliferation, differentiation, and functions. The PD-1/PD-L1 axis on the CD4^+^ T cell surface acts as co-inhibitory molecules for their activation and proliferation (19, 20). In recent years, much attention has been paid to the role of PD-1/PD-L1 pathway in immune evasion within different tumor microenvironments, and PD-1/PD-L1 inhibitors have become crucial therapeutic assets against many cancers. However, there are few studies on the effects and prospects of this inhibitory pathway in the context of autoimmune diseases.

Several studies found that PD-1 is associated with the occurrence and progression of many autoimmune diseases, including type 1 diabetes mellitus and rheumatoid arthritis (21–23). In addition, complex autoimmune diseases, including autoimmune thyroiditis, often occur when PD-1/PD-L1 inhibitors are used in targeted therapy for cancer treatment (24, 25). Thus, decreased PD-1/PD-L1 activity may be one of the pathogenic causes of autoimmune thyroiditis. However, although the contribution of decreased PD-1/PD-L1 activity in non-drug-induced HT might be high, there is no report on potential clinical intervention involving this inhibitory pathway. To evaluate whether the PD-1/PD-L1 pathway could be used as intervention target for HT, we examined the expression of PD-1 and PD-L1 by Th17s and Tregs in HT patients.

## Materials and methods

### Patients, selection criteria, and sample size

This study is a case-control study comparing HT patients (HT group, or case group) and a normal control group (NC group). It was approved by the Medical Ethics Committee of the First Affiliated Hospital of Bengbu Medical College and all patients signed the informed consent form before peripheral blood samples were collected in the endocrinology outpatient.

Recruitment was as follows: 53 patients initially diagnosed with HT based on the criteria of the Chinese Guidelines for Diagnosis and Treatment of Thyroid Disorders were enrolled into the HT group (case group). In addition, 21 age- and sex-matched participants who underwent physical examination in our hospital were recruited for the normal control (NC) group.

The diagnostic criteria for HT were as follows: (1) palpation and thyroid color ultrasonography showed diffuse goiter with a tough texture, especially in patients with enlargement of the vertebral lobe of the thyroid isthmus; (2) patients were positive for thyroid peroxidase antibody (TPOAb) and/or thyroglobulin antibody (TgAb); (3) thyroid fine needle aspiration cytology showed diffuse lymphocytic and plasma cell infiltration, and fibrosis of thyroid gland; (4) thyroid function was normal or TSH increased with or without decreased FT3 and/or FT4. Among these criteria, (1) (2) (4) were necessary conditions.

Exclusion criteria were as follows: (1) Patients with other comorbid autoimmune or inflammatory diseases, such as allergic rhinitis, systemic lupus erythematosus, and ulcerative colitis. (2) Patients with comorbid acute or chronic infectious diseases, such as hepatitis and respiratory tract infection. (3) Patients who recently received thyroid hormone replacement therapy, glucocorticoids, or immunosuppressants. (4) Patients with comorbid malignancy or immunodeficiency. (5) Pregnant or lactating women.

Sample size was determined as follows: decrease in PD-1^+^ Th17 percentage and increase in PD-1^+^ Treg and PD-L1^+^ Treg percentages were the main observation factors suspected to be pathogenic. Based on preliminary results, the mean percentage of PD-1^+^ Th17s in the NC group was 13.41 ± 2.15%, with a standard deviation of 0.73; the percentage of PD-1^+^ Th17s in the HT group was 9.26%, with a standard deviation of 2.77. By setting the level of significance (α; two-tailed) and the power of the test at α = 0.05 and β = 0.10, the PASS V.11 software (NCSS, USA) calculated sample sizes of N1 = 17 and N2 = 34 for the NC and HT groups, respectively. Assuming a non-response rate of the participants of 20%, at least N1 = 17 ÷ 0.8 ≈ 21 and N2 = 34 ÷ 0.8 ≈ 43 participants were required.

### Reagents and equipment

The eBioscience™ Cell Stimulation Cocktail (lot no.: 2046941) was purchased from Thermo Fisher Scientific Inc. (USA). FITC-conjugated anti-human CD4 (lot no.: B262223), PerCP-Cy5.5-conjugated anti-human CD3 (lot no.: B560835), Brilliant Violet 510-conjugated anti-human IFN-γ (lot no.: B323449), PE-conjugated anti-human IL-17A (lot no.: B315520), APC anti-human FOXP3 (lot no.: 1995356), PE-conjugated anti-human CD25 (lot no.: 2173867), Brilliant Violet 421-conjugated anti-human CD274 (lot no.: B320732), PE/Cyanine7-conjugated anti-human CD279 (lot no.: B318888), Fixation/Permeabilization Concentrate (lot no.: 2084746), eBioscience™ Fixation/Perm Diluent (lot no.: 2047346), Permeabilization Buffer 10× (lot no.: 2060497), and FIX&PERM (Fixation Medium A, Permeabilization Medium B) (lot no.: 17159) were purchased from Biolegend (USA).

### PBMC processing and staining

CD4^+^CD3^+^IL-17^+^IFN-γ^−^ Th17 staining was performed as follows: 2 mL of fasting (≥ 8 h) peripheral blood from the participants was collected into a vacutainer containing 0.2 ml heparin sodium. An aliquot of 250 μL was transferred to a new tube containing an equal volume of RPMI 1640 culture medium. Two microliters of Cell Stimulation Cocktail were added and mixed evenly, following with 4 h incubation at 37°C with 5% CO_2_. After stimulation, 200 μl of the cell mixture was transferred to a new tube (cell concentration: 1×10^6^/mL) and incubated for 15 min in the dark with 50 µg/mL of each of the following antibodies: FITC-conjugated anti-CD4, PerCP-Cy5.5-conjugated anti-CD3, BV421-conjugated anti-CD274, and PE/Cy7-conjugated anti-CD279. Following surface staining, 2 mL of diluted hemolysin was added to the cells and incubated in the dark for 10 min. After lysis the cells were centrifuged at 300 × g and room temperature for 5 min. The supernatant was discarded, and the cells were fixed in 100 μL of Fixation Medium A for 5 min at room temperature. The cells were washed by addition of 2 mL of phosphate buffer saline (PBS) and centrifugation at 300 × g for 5 min. The cells were permeabilized with 100 μL of Permeabilization Medium B at room temperature in the dark for 5 min. The intracellular staining was performed by adding 5 μL each of BV510-conjugated anti-IFN-γ and PE-conjugated anti-IL-17A in the dark for 15 min. The cells were washed by addition of 2 mL of PBS and centrifugation at 300 × g for 5 min. Finally, the cells were resuspended in 0.5 mL of PBS and analyzed on a flow cytometer.

CD4^+^CD25^+^FOXP3^+^ Treg staining was performed as follows: 200 μL whole blood prepared as above was transferred into a new tube and was incubated with 50 µg/mL of each of the following antibodies for 15 min in the dark: FITC-conjugated anti-CD4, BV421-conjugated anti-CD274, and PE/Cy7-conjugated anti-CD279. The red blood cells were lysed with 2 mL of diluted hemolysin in the dark for 10 min. The PBMC were harvested by centrifugation at 300 × g at room temperature for 5 min. They were resuspended in 1 mL of membrane permeabilization and fixation working solution and incubated in the dark for 40 min. After centrifugation at 300 × g for 5 min, the cells were washed twice with 2 mL of washing solution (Permeabilization Buffer and distilled water at 1:9 ratio). Finally, the cells were resuspended in 2 mL of washing solution and incubated with 5 μL of APC-conjugated anti-FOXP3 antibody in the dark for 40 min. The cells were washed using washing buffer before being resuspended in 0.5 mL of PBS for acquisition of the sample on a flow cytometer.

Each experiment included experimental tubes stained with the specific antibodies described above, and isotype control (ISO) tubes stained with fluorochrome-conjugated antibody of matching isotypes and of irrelevant specificity in human, used at the same concentrations as the corresponding specific antibodies. Single-stained tubes covering all used fluorochromes were used as compensation controls.

### Assessment of the thyroid functions

Serum markers of thyroid function (TSH, FT_3,_ FT_4,_ TT_3_ and TT_4_) and TPOAb and TgAb autoantibodies in the different groups were measured by chemiluminescence. Reference ranges were as follow: TT_3_, 1.01–2.95 nmol/L; TT_4_, 55.34–160.88 nmol/L; FT_3_, 2.77–6.5 pmol/L; FT_4_, 10.43–24.32 pmol/L; TSH, 0.4–4.34 mIU/L; TPOAb, < 60 U/mL; TgAb, < 60 U/mL.

### Statistical methods

The statistical analyses were performed using SPSS 26.0 software. The chi-squared test was used for qualitative data. The quantitative data are expressed as mean ± standard deviation when normally distributed with homogeneous variance. Independent sample t-test was used for inter-group comparison and Pearson test was used for correlation analysis. Non-parametric tests were used for skewed distribution data or data with non-homogeneous variance, in which case the results are expressed as Z. Spearman test was used for correlation analysis.

## Results

### Comparison of the clinical data from the HT and NC groups

In total, 53 HT patients, including one male and 52 female, and 21 NC, including two male and 19 female were recruited. The age range of the participants was 15–66 years. There were no significant differences in gender and age between the two groups (P > 0.05). While there were no significant differences in FT_3_ and FT_4_ levels between the two groups (P > 0.05), TSH level (P < 0.05), and TPOAb and TgAb levels (P < 0.001) were significantly different between the two groups. The data on age and thyroid function markers were normally distributed or approximately normally distributed in the two groups (**Table 1**).

**Table 1:** Comparison of the clinical data from the HT and the NC group.

	HT (n = 53)	NC (n = 21)	t-test/X^2^ test	
			t/X^2^	P-value
Gender (Male/Female)	1/53	2/21	2.255	0.133
Age (years)	38.91 ± 13.02	38.57 ± 14.52	0.096	0.924
FT_3_ (pmol/L)	4.50 ± 0.94	4.73 ± 0.44	−1.091	0.279
FT_4_ (pmol/L)	15.07 ± 4.69	15.60 ± 2.19	−0.654	0.515
TSH (mIU/L)	8.06 ± 17.87	2.29 ± 1.12	2.320	0.024
TPOAb (U/mL)	711.0 ± 576.51	56.59 ± 9.75	8.262	< 0.001
TgAb (U/mL)	340.2 ± 223.02	33.04 ± 13.84	9.979	< 0.001

The numbers represent mean ± standard deviation. HT, Hashimoto’s thyroiditis; NC, normal control.

### HT patients have a higher percentage of peripheral blood Th17s and a lower percentage of peripheral blood Tregs than the healthy individuals

**Figure 1:**
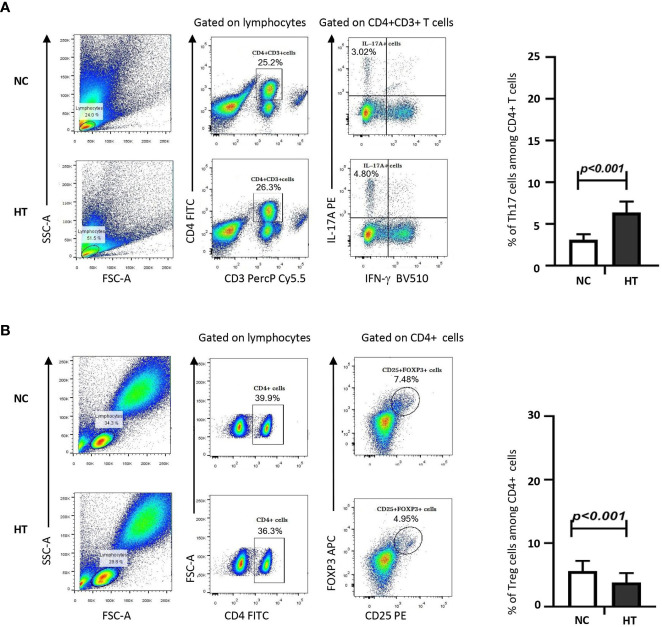
Peripheral blood Th17s were increased, whereas peripheral blood Tregs were decreased in HT patients compared with NC. The percentages of IL-17^+^ Th17s **(A)** or CD25^+^FOXP3^+^ Tregs **(B)** among the peripheral blood CD4^+^ T cells of NC and HT patients was quantified by flow cytometric analysis. The gating strategy is shown on representative dot plots from a NC and a HT patient. The bar charts on the left-hand side summarize the data obtained for the NC (n = 21) and the HT (n = 53) groups. Differences between groups were assessed by t-test and the p-values are indicated.

The average percentage of Th17s among the peripheral blood CD4^+^ T cells of the HT group was significantly higher compared with that of the NC group [(6.38 ± 1.32)% versus (3.12 ± 0.66)%; t = 14.110, P < 0.001] **(** **Figure 1A**; **Table 2**). Conversely, the percentage of Tregs among the peripheral blood CD_4_^+^ T cells of the HT group was significantly lower than that of the NC group [(3.82 ± 1.48)% versus (5.61 ± 1.60)%; t = −4.599, P < 0.001] (**Figure 1B**; **Table 2**).

**Table 2:** Percentages of the peripheral blood T cell subsets statistically different between the HT and the NC groups.

T cell phenotype	HT group (n = 53)	NC group (n = 21)	t-test	
			t-value	P-value
Th17	6.38 ± 1.32	3.12 ± 0.66	14.110	< 0.001
PD-1^+^ Th17	6.46 ± 2.77	18.51 ± 3.96	−14.842	< 0.001
Treg	3.82 ± 1.48	5.61 ± 1.60	−4.599	< 0.001
PD-1^+^ Treg	17.01 ± 3.04	10.23 ± 2.77	8.850	< 0.001
PD-L1^+^ Treg	16.60 ± 9.58	11.36 ± 10.14	2.089	< 0.05

The numbers represent the percentage of cells of a given phenotype expressed as mean ± standard deviation. HT, Hashimoto’s thyroiditis; NC, normal control.

### HT patients’ Th17s express lower levels of the checkpoint molecule PD-1 than their counterparts in healthy individuals

**Figure 2:**
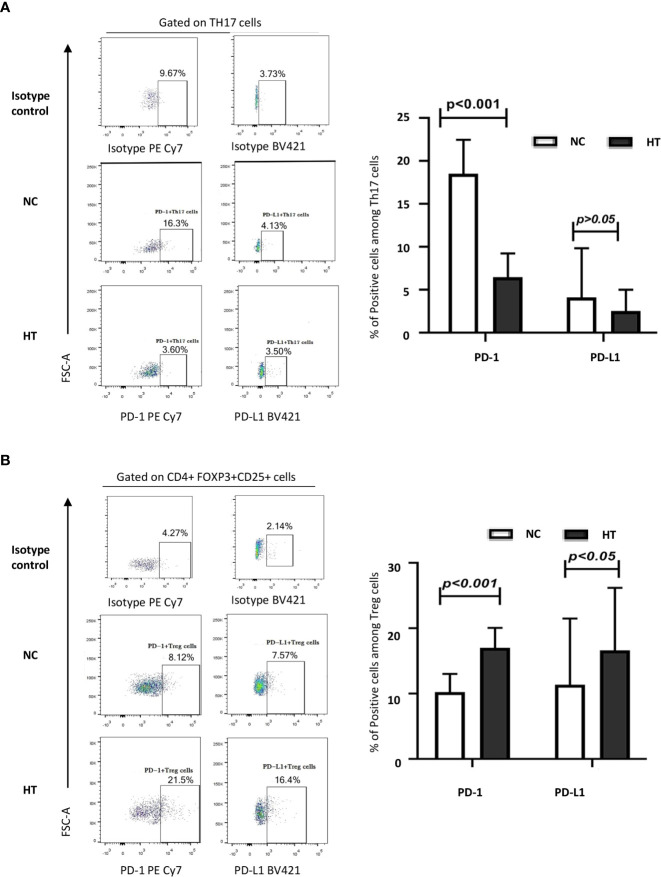
Comparison of the expression of PD-1 and PD-L1 on peripheral blood Th17s and Tregs from NC and HT patients. The percentages of IL-17^+^ Th17s expressing PD-1 and PD-L1 **(A)**, and the percentages of CD25^+^FOXP3^+^ Tregs expressing PD-1 and PD-L1 **(B)** among the peripheral blood CD4 T cells of NC and HT patients was quantified by flow cytometric analysis. Representative dot plots display PD-1 and PD-L1 positive cells based on negative controls using isotype control antibodies. The bar charts on the left-hand side summarize the data obtained for the NC (n = 21) and the HT (n = 53) groups. Differences between groups were assessed by t-test and the p-values are indicated.

The percentage of peripheral blood Th17s expressing PD-1 was significantly lower in the HT patients than in the NC group [(6.46 ± 2.77)% vs (18.51 ± 3.96)%; t = −14.842, P < 0.001]. However, there was no difference in the percentage of peripheral blood PD-L1^+^ Th17s in in the HT and NC groups (P > 0.05) (**Figure 2A**; **Table 2**).

### The lower percentage of peripheral blood Tregs in HT patients is accompanied by a higher proportion of these cells expressing PD-1 and PD-L1, than in NC

The peripheral blood Tregs from the HT patients expressed PD-1 at a significantly higher frequency than those from the NC group [(17.01 ± 3.04)% versus (10.23 ± 2.77)%; t = 8.850, P < 0.001] (**Figure 2B** **;** **Table 2**). Similarly, the HT patients had a significantly higher frequency of peripheral blood PD-L1^+^ Tregs than the NC [(16.60 ± 9.58)% versus (11.36 ± 10.14)%; t = 2.089, P < 0.005] (**Figure 2B**; **Table 2**).

## Discussion

HT pathology is characterized by lymphocyte infiltration of the thyroid tissues, fibrosis, parenchymal atrophy, and eosinophilic changes in some of the thyroid follicular cells. This complex autoimmune pathology involves various immune cells and molecules, including T and B cells, macrophages, natural killer cells, various cytokines, and autoantibodies. A previous study (26) found that FOXP3 mRNA expression was significantly reduced in the PBMCs from HT patients, while the number of peripheral blood Th17s and IL-17 mRNA expression in HT thyroid tissues were increased. These observations suggest that alterations of Th17 and Treg number and functions may play an important role in HT. The significance of the Th17/Treg ratio in HT has been confirmed by a large volume of evidence (27, 28).

PD-1/PD-L1 has been found to operate on Tregs (29) and Th17s (30) in a broad range of diseases and experimental setups. In addition, the pattern of PD-1/PD-L1 expression is altered in HT (31). Yet, the specific activity of this molecular pathway on Th17s and Tregs during HT remains to be elucidated. Therefore, we sought to characterize the changes in PD-1 and PD-L1 expression on the surface of Th17s and Tregs in peripheral blood from HT patients to gain preliminarily insights into a potentially aberrant activity of the PD-1/PD-L1 pathway on Th17/Treg differentiation and balance.

Consistent with previous studies (26, 32), the percentage of Tregs decreased and the percentage of Th17s increased in HT (P < 0.001). Xue, Yu (32) found that the Treg/Th17 balance during HT occurrence and progression was biased towards Th17s, and the number of Tregs was negatively correlated with thyroid-specific autoantibody and TSH levels. Therefore, the current study consolidates the hypothesis that imbalance in Th17/Treg percentage is closely associated with HT pathogenesis.

Our study found that the increase in Th17 proportion was accompanied by a lower proportion of these cells expressing PD-1 (P < 0.001). This result is consistent with a possible loss of a negative regulation exerted by PD-L1^+^ antigen-presenting cells on Th17 activity through PD-1 (29, 33, 34). PD-1 diminution on Th17 could originate or be a consequence of the proinflammatory and pathogenic activity of Th17s in HT (35). The exact significance of the changes in PD-1 expression on Th17s’ ability to cause immune injury by secreting pro-inflammatory cytokines during HT needs further investigation.

In addition, we showed that although the proportion of Tregs was decreased in HT patients, these cells expressed PD-1 and PD-L1 at higher frequencies. This increase could result in an auto-inhibition of Treg proliferation through a negative signal mediated by PD-1/PD-L1, disrupt immune tolerance, and ultimately promote HT (36). T cell receptor (TCR) is activated by antigen-antibody binding in the immune response to abnormally exposed thyroid autoantigens. The binding of PD-1 to PD-L1 on Tregs may result in dephosphorylation of CD28 and CD226 and partial dephosphorylation of TCR on T cell membrane by recruiting phosphatase SHP1/SHP2, which reduces the activation of the TCR pathway. This reduces the expression of transcription factors and ultimately inhibits the proliferation of Treg cells and reduces immune tolerance (37). However, the effect of the PD-1/PD-L1 pathway in Tregs varies depending on the disease contexts and experimental setups. Francisco, Salinas (38) found that PD-1 and PD-L1 could enhance FOXP3 expression in induced Treg, promote their differentiation, and enhance their anti-inflammatory effects. Further, Ramos-Casals, Brahmer (39) showed that PD-L1 could inhibit Akt/mTOR signaling pathway and induce the conversion of effector CD4^+^ T cells into Tregs. These studies support that a higher proportion of Tregs expressing PD-1 and PD-L1 could be beneficial in HT and might result from compensatory regulatory mechanisms operating to counteract autoimmune inflammation. However, in other pathological contexts, PD-1/PD-L1 axis may repress Treg functions, including during infection (40) or in hyper-progressive tumors resistant to checkpoint inhibitor therapy targeting PD-1 (41). These contradictory data may explain that, despite higher frequency of PD-1^+^ and PD-L1^+^ Tregs in HT patients, these cells are inefficient in controlling ongoing autoimmunity.

Thus, the complexity of the immune mechanisms at play in HT and this first report on a potential role of PD-1/PD-L1 in the CD4^+^ T cell dysfunction underlying HT call for further in-depth studies involving a large sample size and functional approaches to elucidate the exact implication of this pathway in HT pathogenesis.

In the tumor microenvironment, PD-L1 on tumor cells can bind to PD-1 on T cells, trigger downstream inhibitory signals of TCR, and inhibit the immune response (42). PD-1/PD-L1 immune checkpoint inhibitors are successfully used in treatments for many types of cancer types (43, 44), but autoimmune diseases such as autoimmune thyroiditis occur from time to time. Based on our understanding, there is no study that specifically covers how PD-1/PD-L1 affects the differentiation and function of CD4^+^T cells in the pathological mechanism of HT. Determining the relationship between the PD-1/PD-L1 pathway and the expression of decisive transcription factors such as ROR γ t and Foxp3 in Th17 and Treg differentiation, will be an important direction for future future. In addition, selectively changing the expression of PD-1/PD-L1 in immune cells has become a new direction for the treatment of autoimmune diseases. Researchers have applied IFN-γ induction technique to increase the expression of PD-L1 on macrophages, so they bind to PD-1 on the surface of CD4^+^ T cells and inhibit the immune activity of CD4^+^ T cells (45). In this work, we report preliminary findings of the abnormal PD-1/PD-L1 pathway activity in HT. Thus, the induction of these molecules on the CD4^+^ T cell surface is theoretically possible (46), and increasing PD-1/PD-L1 expression on the CD4^+^ T cells infiltrating the thyroid microenvironment could decrease Th17 activity and increase Treg proliferation and tolerogenic functions. This strategy could alleviate or reverse inflammatory damage in HT. However, increasing the expression of PD-1/PD-L1 on immune cells and ensuring basic immune function to prevent chronic infection, tumors, and other diseases poses a serious challenge that must be overcome in the future.

## Conclusion

The abnormality in the PD-1/PD-L1 pathway activity specifically the increased percentage of Th17s and decreased percentage of PD-1^+^Th17s in the HT group, suggests that a loss of control on Th17 activity through the checkpoint inhibitory axis PD-1/PD-L1 may participate in disease pathogenesis. While the decreased percentage of Tregs in HT patients may explain a lack of regulatory functions able to prevent the autoimmune destruction of the thyroid, the significance of the increased frequency of Tregs expressing PD-1 and PD-L1, previously reported to boost Tregs differentiation, remains to be established. Elucidating this apparent contradiction may reveal important mechanisms underlying HT pathogenesis.

## Data availability statement

The datasets presented in this study can be found in online repositories. The names of the repository/repositories and accession number(s) can be found in the article/**Supplementary Material**.

## Ethics statement

The studies involving human participants were reviewed and approved by EC of the First Affiliated Hospital of Bengbu Medical College; The First Affiliated Hospital of Bengbu Medical College, Bengbu, Anhui, P.R. China. Written informed consent to participate in this study was provided by the participants’ legal guardian/next of kin.

## Author contributions

JF and GJ conceived and designed the study, QW collected date and performed the statistical analysis. JF wrote the first draft which was revised by GJ and XP. The study was supervised by LZ and LY. All the authors contributed to the study and approved the submitted version.

## Funding

Major Program of Nature Science Foundation of Anhui Higher Education Institutions China (KJ2019ZD29); High Level Scientific and Technological Innovation Team Project of the First Affiliated Hospital of Bengbu Medical College (BYYFY2022TD001); Science and Technology Development Fund Project of Bengbu Medical College(BYKF1862); and Major Program of Nature Science Foundation of Bengbu Medical College(BYKY1839ZD).

## Acknowledgments

We would like to thank all participants involved in this study.

## Conflict of interest

The authors declare that the research was conducted in the absence of any commercial or financial relationships that could be construed as a potential conflict of interest.

## Publisher’s note

All claims expressed in this article are solely those of the authors and do not necessarily represent those of their affiliated organizations, or those of the publisher, the editors and the reviewers. Any product that may be evaluated in this article, or claim that may be made by its manufacturer, is not guaranteed or endorsed by the publisher.
